# Postoperative radiotherapy for supraglottic cancer on real-world data: can we reduce dose to lymph node levels?

**DOI:** 10.1186/s13014-023-02228-1

**Published:** 2023-02-22

**Authors:** Yi Xu, Yumei Wei, Jingbo Wang, Jianghu Zhang, Xuesong Chen, Runye Wu, Qingfeng Liu, Yuan Qu, Kai Wang, Xiaodong Huang, Jingwei Luo, Wei Xu, Ye Zhang, Junlin Yi

**Affiliations:** 1 Department of Radiation Oncology, National Cancer Center/National Clinical Research Center for Cancer/Cancer Hospital, Chinese Academy of Medical Sciences (CAMS) and Peking Union Medical College (PUMC), No17, Panjiayuan Nanli, Chaoyang District, Beijing, 100021 People’s Republic of China; 2 Department of Radiation Oncology, National Cancer Center/National Clinical Research Center for Cancer/Hebei Cancer Hospital, Chinese Academy of Medical Sciences, Langfang, 065001 People’s Republic of China; 3 Department of Head and Neck Radiotherapy, Shandong Provincial ENT Hospital, Cheeloo College of Medicine, Shandong University, Jinan, People’s Republic of China; 4 Department of Head and Neck Surgery, Shandong Provincial ENT Hospital, Cheeloo College of Medicine, Shandong University, Jinan, 250022 People’s Republic of China

**Keywords:** Dose reduction, Postoperative radiotherapy, Supraglottic cancer

## Abstract

**Purpose:**

To evaluate prognosis for reducing postoperative radiotherapy (PORT) dose to lymph node levels of supraglottic cancer (SC) on real-world data.

**Method and materials:**

Patients were derived from two cancer centers. In center 1, the involved nodal levels (high-risk levels, HRL) and the next level received a dose of 60.06 Gy/1.82 Gy per fraction, while the other uninvolved levels (low-risk levels, LRL) received 50.96 Gy/1.82 Gy per fraction. In center 2, all received 50 Gy/2 Gy per fraction. The rates of high-risk levels control (HRC), regional control (RC), overall survival (OS), progression-free survival (PFS) and distant metastasis-free survival (DMFS) were calculated by Kaplan–Meier method.

**Result:**

Totally, 124 patients were included (62 in center 1, 62 in center 2). Most patients (106, 85.5%) had a stage T3/N + tumor. The median follow-up was 45 months (range 1–163 months). There were no significant differences in terms of OS (p = 0.126), RC (p = 0.514), PFS (p = 0.195) and DMFS (p = 0.834). Most regional recurrences (4, 80%) occurred within three years of treatment, and all occurred within the target volumes. No regional failure occurred in HRL in center 1, while three (3/4) failures occurred in center 2. Dose reduction prescription to HRL led to a lower HRC rate (100% vs. 90.6%, p = 0.009). While the rates of LRL control (98.4%) were equal between the two centers.

**Conclusion:**

Compared with a standard dose, the reduced dose to involved nodal levels showed inferior regional control for PORT, while uninvolved nodal levels showed equal outcomes. A dose of 50 Gy for HRL may be an unfavorable treatment option for SC.

## Introduction

Postoperative radiotherapy is the standard treatment for locally advanced head and neck cancers in patients with adverse risk factors. The US Intergroup trial (RTOG 9501) and the European trial (EORTC 22,931) further confirmed that postoperative concurrent chemoradiotherapy significantly improves locoregional control and disease-free survival of patients with high-risk adverse factors [1, 2]. In the postoperative setting, all pathologically involved nodal levels (high-risk levels, HRL) are supposed to receive a dose of 57–60 Gy in 30 fractions, and uninvolved nodal levels (low-risk levels, LRL) receive 50 Gy in 25 fractions [3]. However, this treatment paradigm is primarily based on two-dimensional plans. Considering the advancement in radiotherapy and a more accurate staging method, lower doses to cervical lymph node levels may be acceptable.

A dose of 50–60 Gy is considered to sterilize micrometastatic nodal disease, as reported in previous studies [4]. Withers pointed out that doses of 50 Gy in 2-Gy fractions could achieve an overall 90% reduction in the incidence of metastases [5]. As previously reported, doses of 50 Gy can effectively control minute deposits. However, in many modern trials, a prescription of 60 Gy is usually delivered to the HRL and 50 Gy to the LRL. Most recurrence events occur in the primary tumor field in patients with laryngeal cancer, while solitary regional failures are rare [6, 7]. Thus, the lower doses administered to HRL may be sufficient, particularly when combined with concurrent systemic therapies.

In addition, the delivery of low radiation doses to the neck decreases acute and late toxicity, protecting normal tissue. In a few published reports, the radiation dose delivered to the pharyngeal constrictor muscles predicted the risk of pharyngeal dysphagia [8–10]. A multicenter prospective randomized clinical trial found that reduction in the radiation dose delivered to LRL from 50 to 40 Gy resulted in a trend toward less dysphagia at 6 months without compromising tumor control [11, 12]. In recent years, radiation oncologists have identified radiation dose reduction as a factor that can improve the quality of life of patients.

Therefore, we retrospectively analyzed two oncology centers’ real-world data to determine whether a reduction in radiation doses delivered to postoperatively involved lymph nodes was reasonable. High-risk level control (HRC) was the primary evaluation parameter for the efficacy and safety of the de-intensification regimen.

## Method and materials

### Patient selection

Patient data were derived from two centers at two hospitals: center 1 at hospital A, between January 2007 and December 2017, and center 2 at hospital B between January 2013 and December 2018. Patients with histologically confirmed supraglottic squamous cell carcinoma treated with primary site surgery, selective lymph node dissection, and postoperative intensity-modulated radiotherapy (IMRT) were enrolled. Eligible patients were reclassified according to the American Joint Committee on Cancer pathological staging manual, 8th edition. Exclusion criteria included other head and neck cancers, developed recurrence or distant metastases before postoperative radiotherapy (PORT), received any neoadjuvant treatment, and history of neck radiotherapy or surgery.

Patients were required to undergo computed tomography (CT) and/or magnetic resonance imaging (MRI) of the head and neck, and laryngoscopy before surgery and PORT. Postoperative pathological reports included data on the number of total and positive lymph nodes, tumor differentiation, extranodal extension (ENE), surgical margin status, and type of operation.

### Target delineation and dose prescription

Primary gross tumor volume (GTVtb) was defined as the primary tumor bed with the aid of preoperative diagnostic imaging, operative findings, and pathology reports. It should be edited based on anatomical changes caused by surgery [3]. In center 1, clinical target volume (CTV) 1 included the GTVtb with a margin of 1–1.5 cm, for all HRL and some LRL. It also covered a 5-mm safety margin around the positive nodes with ENE. For patients with stage T3–4N0 tumors, CTV1 included bilateral levels II–III. CTV2 included the remainder of the LRL. In center 2, CTV included GTVtb with a margin of 1–1.5 cm and the HRL and LRL. All were edited for anatomical barriers, and detailed information on contouring elective nodal levels are shown in the Supplementary Material. The planning target volume (PTV) was defined by providing an isotropic margin of 3–5 mm around the GTVtb and each CTV. All lymph nodes with ENE were delineated under GTVnd-tb (tumor bed for lymph nodes with ENE) according to the preoperative CT or MRI images.

In both centers, the PTV associated with the GTVtb (PGTVtb) (only in the case of a positive margin) and GTVnd-tb (lymph nodes with ENE) received a dose of 66–70 Gy. In center 1, the PTV associated with CTV1 (PTV1) received a dose of 60.06 Gy in 33 fractions, and CTV2 (PTV2) received a dose of 50.96 Gy in 28 fractions. In center 2, PTV received a dose of 50 Gy in 25 fractions of 2 Gy each (Table 1).

**Table 1:** Selection of target volumes for supraglottic cancer

Center 1		
Stage (AJCC/UICC 8th)	CTV1	CTV2
T3-4N0	GTVtb with a margin of 1–1.5 cm and bilateral levels II, III	Bilateral levels IV
N +	GTVtb with a margin of 1–1.5 cm and involved nodal levels and the next levels	Uninvolved bilateral levels II-IV; Ib or/and V
Dose prescription	60.06 Gy in 33 fractions	50.96 Gy in 28 fractions
Center 2		
Stage (AJCC/UICC 8th)	CTV	
N0/N +	GTVtb with a margin of 1–1.5 cm and bilateral levels II-IV	
Dose prescription	50 Gy in 25 fractions	
ENE or positive margin		
	GTVnd-tb, GTVtb	
Dose prescription	66–70 Gy	

*AJCC*, American Joint Committee on Cancer; *UICC*, Union for International Cancer

Control; *ENE*, extra nodal extension; *CTV*, nodal clinical target volume

### Concurrent chemotherapy

Patients with ENE and/or positive resection margins received cisplatin in addition to PORT. Concurrent chemotherapy consisted of 100 mg/m^2^ cisplatin every 3 weeks for 2–3 cycles or 40 mg/m^2^ each week for 5–7 cycles.

### Follow-up and statistical analysis

Follow-up visits were advised every 3 months for the initial 2 years after treatment, every 6 months for 3 years, and yearly thereafter. Routine assessment included clinical examination, laryngoscopy, and radionic imaging.

The chi-square test and t-test were used to compare differences in patient characteristics between the two centers. The primary outcome measure was the HRC calculated from the first day of surgery until regional recurrence using the Kaplan–Meier method. Additionally, recurrent lymph nodes were defined based on CT/MRI, which was co-registered with images of the IMRT-planning CT. To evaluate failure patterns, we classified the HRC as having no regional failure within the HRL. Other endpoints, such as overall survival (OS), regional control (RC), progression-free survival (PFS), and distant metastasis-free survival (DMFS), were also calculated from the first day of surgery until the events occurred by using the Kaplan–Meier product limit test. Patients who did not experience any of these events were censored at the time of the last follow-up. The statistical significance of the differences between the two centers was evaluated using the log-rank test. Statistical analyses were performed with SPSS version 26 (SPSS Inc., Chicago, IL, USA).

## Results

Overall, 124 patients were included from the two centers (62 in center 1 and 62 in center 2), all of whom received IMRT after surgery. The patients’ essential characteristics and treatment details are shown in Table 2. More than 90% of the patients were men with a high smoking index (≥ 400). Moderate differentiation was observed in most patients in both centers. Most patients (106, 85.5%) had stage T3/N + tumors, whereas more patients had stage T2N0 tumors in center 2 than in center 1 (27.4% vs. 1.6%). All patients were treated with selective neck dissection, and 91.9% underwent bilateral neck dissection. A few patients who underwent ipsilateral neck dissection for lateralized lesions were from center 1 (n = 10, 16.1%). A total of 5944 lymph nodes were resected, and the average number of dissected lymph nodes was 48 per person. Only two patients had lymph node counts less than 18. The median number of positive lymph nodes was two per person (range 0–26). Nearly half of the patients in center 1 were in the N2 stage, while those in center 2 were in the N0 stage. Concurrent chemotherapy with cisplatin (generally 2–3 cycles) was administered to 43.5% of the patients in center 1 and to 11.3% of the patients in center 2. Meanwhile, the ENE rates were 21% and 14.5% in centers 1 and 2, respectively.

**Table 2:** Patient characteristics

Characteristics	Center 1 n (%)	Center 2 n (%)	P
Gender			0.243
Male	60(96.7)	57(91.9)	
Female	2(3.3)	5(8.1)	
Age (y)			0.104
Median; Range	58; 42–75	64; 34–79	
Smoking index			0.442
Median; Range	600; 0–2000	800; 0–2400	
Differentiation			0.288
Well	2 (3.2)	3 (6.4)	
Moderate	37 (59.7)	44 (71)	
Poor	23 (37.1)	15 (24.2)	
Extra nodal extension			0.347
Yes	13 (21.0)	9 (14.5)	
No	49 (79.0)	53 (85.5)	
T stage			0.002*
T1	3 (4.8)	0 (0)	
T2	9 (14.5)	27 (43.5)	
T3	40 (64.5)	32 (51.6)	
T4	10 (16.1)	3 (4.8)	
N stage			0.004*
N0	11 (17.7)	30 (48.4)	
N1	8 (12.9)	5 (8.1)	
N2	31 (50)	21 (33.8)	
N3	12 (19.4)	6 (9.7)	
Stage (UICC 2017)			< 0.001*
II	1 (1.6)	17 (27.4)	
III	14 (22.6)	17 (27.4)	
IVA	34 (54.8)	22 (35.5)	
IVB	13 (21)	6 (9.7)	
Concurrent chemotherapy			
Yes	27 (43.5)	7 (11.3)	< 0.001*
No	35 (56.5)	55 (88.7)	
CDDP prescription			< 0.001*
100 mg/m2 every 3 weeks	25	7	
− 40 mg/m2 each week	2	0	
Neck dissection			0.001*
Ipsilateral	10 (16.1)	0 (0)	
Bilateral	52 (83.9)	62 (100)	
Second primary cancer	13 (21)	8 (12.9)	0.231
Total	62	62	NA

NA, not available. *p < 0.05

**Fig. 1:**
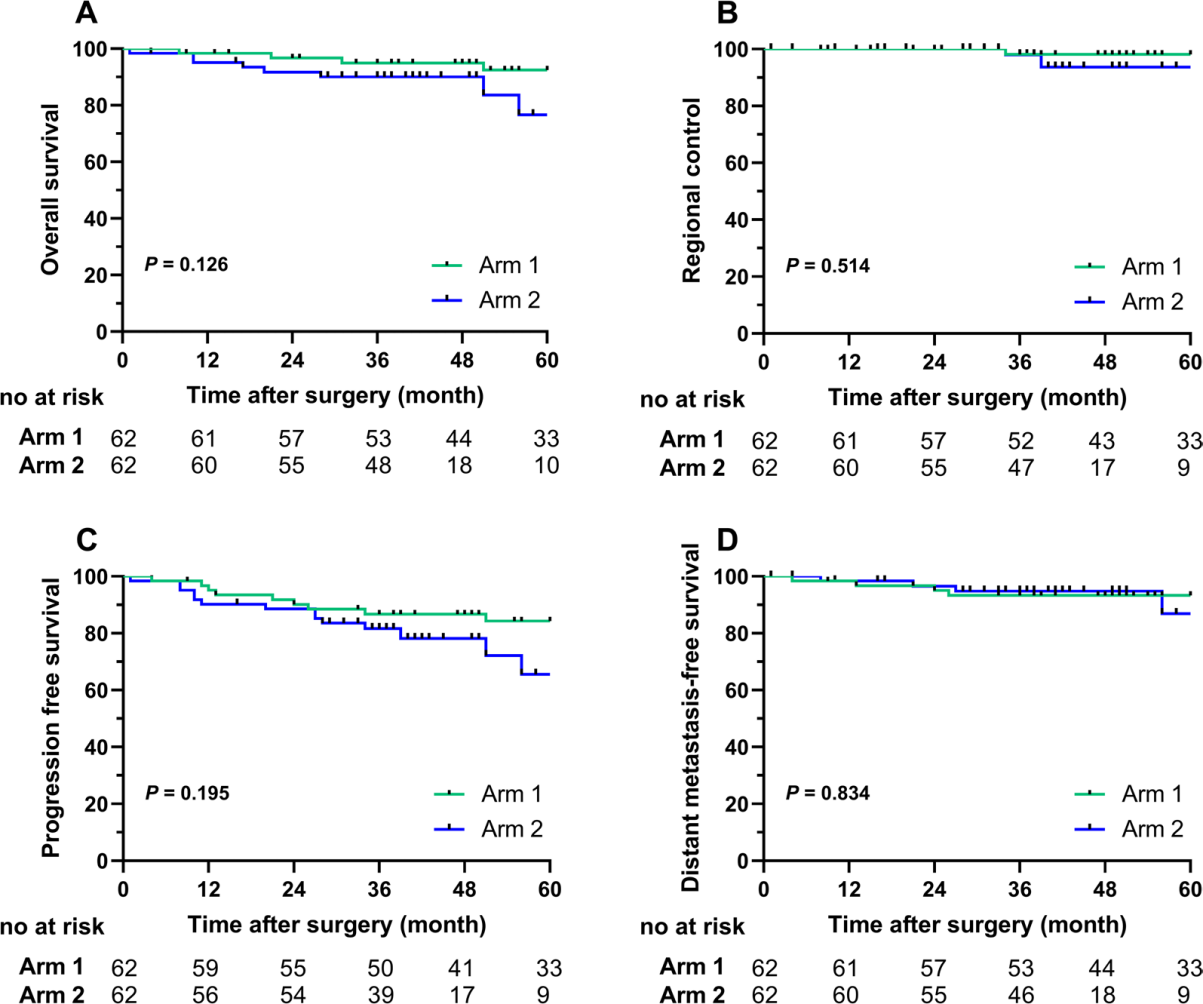
The comparison of the treatment results between center 1 and center 2

The median follow-up period was 45 months (range 1–163 months) for the entire group, with 62 months (range 8–163 months) for center 1 and 37 months (range 1–84 months) for center 2. The 3-year cumulative RC rates were 100% in center 1 and 96.6% in center 2 without a significant difference (p = 0.149). The OS (p = 0.126), RC (p = 0.514), PFS (p = 0.195), and DMFS (p = 0.834) outcomes were similar between patients in the two centers (Fig. 1). The estimated 3-year OS rates were 92.4% and 90%, 3-year RC rates were 91.3% and 90.6%, 3-year PFS rates were 84.3% and 81.7%, and 3-year DMFS rates were 93.3% and 94.8% in centers 1 and 2, respectively. A total of 21 (16.9%) patients developed second primary tumors (21% in center 1, 12.9% in center 2) that were mainly located in the lungs (9, 42.8%).

### Patterns of failure

**Fig. 2:**
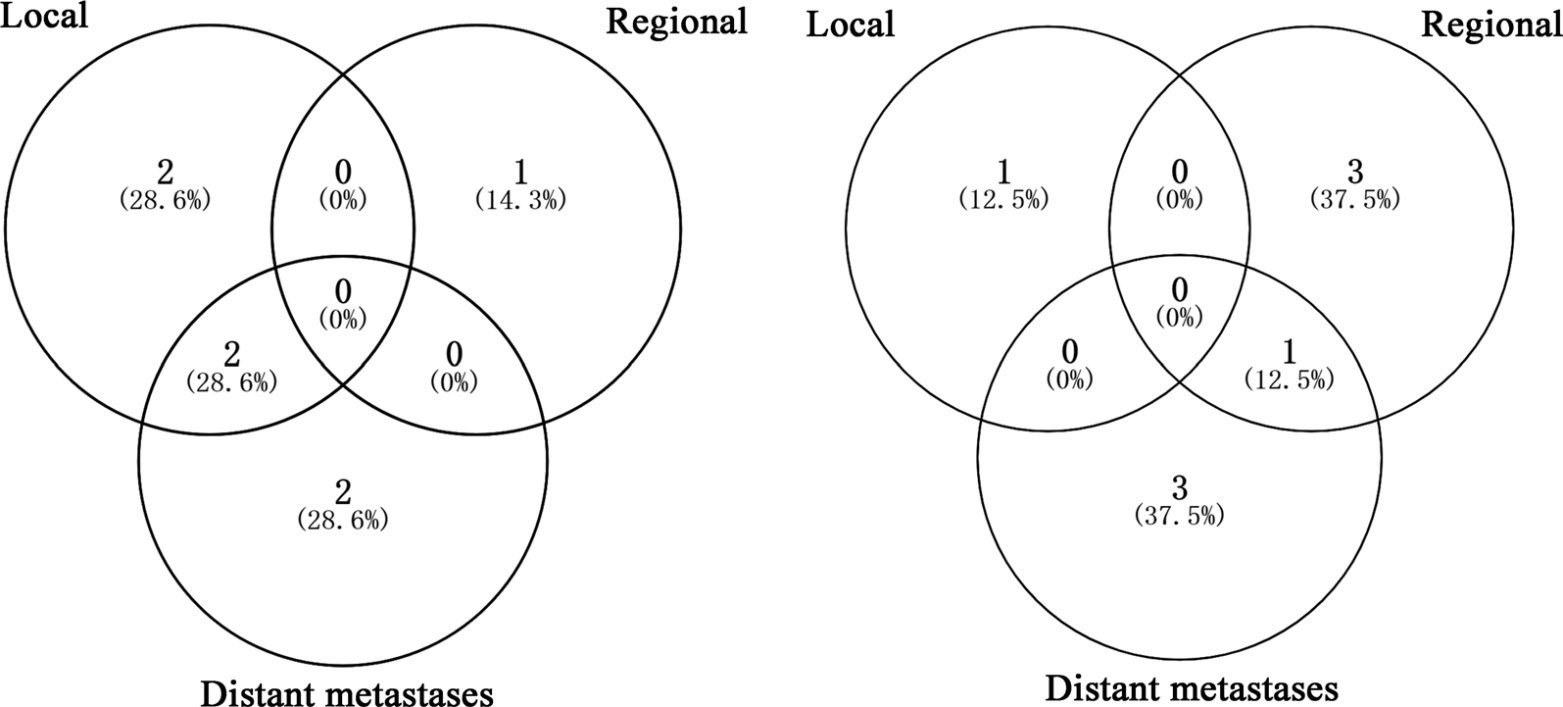
Venn’s circles represent the pattern of failure

Five patients developed regional recurrence, with four patients having regional recurrence alone, and one patient having regional recurrence accompanied by distant metastasis. Five patients had distant metastases alone, and three had local recurrence only for the first failure (Fig. 2). Overall, most local–regional recurrence events (9/10) occurred within 2 years of treatment.

**Fig. 3:**
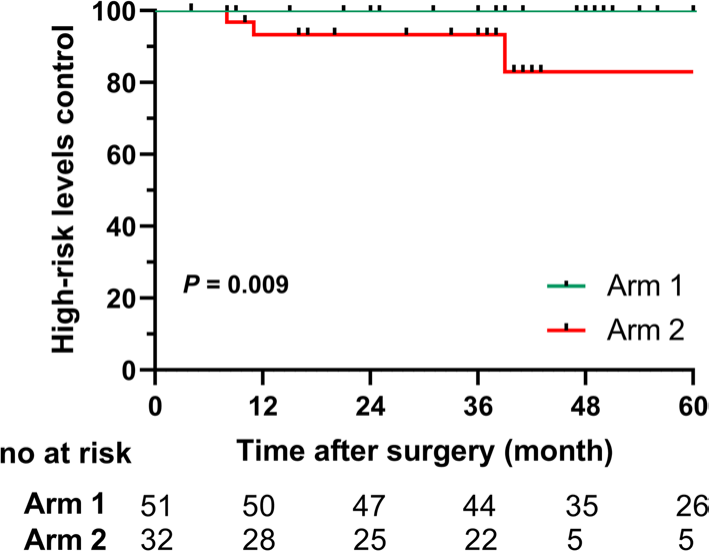
The comparison of the high-risk levels control between center 1 and center 2

The primary characteristics of patients with regional lymph node recurrence are shown in Table 3. By analyzing the diagnostic MRI or CT performed at the time of recurrence, the distributions of different types of regional recurrence in the two centers were identified. All regional failures occurred in patients with pathologically positive lymph node levels and within target volumes. In center 1, one patient treated with ipsilateral neck dissection experienced contralateral neck failure located in the LRL. In center 2, four patients experienced regional recurrence, and three of events were within the HRL. The HRC rate was significantly higher in center 1 (100% vs. 90.6%, p = 0.009; Fig. 3). In comparison, the control rates of LRL (98.4%) were equal between the two centers.

**Table 3:** Basic characteristics of patients with regional failure

Patient no	Centers	Tumor location	TNM stage	Neck dissection	Dose(Gy)/fraction	Recurrence region	Time to recurrence (months)	Results
1	Center 1	unilateral without midline involvement	T2N2b	Ipsilateral level II-IV neck dissection	50–60/25–30	Contralateral level II (LRR)	34	Complicated with base of tongue cancer, 40 months later
2	Center 2	central	T2N2c	Bilateral level II-IV neck dissection	50/25	Level IV of left neck (LRR)	34	Survival
3	Center 2	unilateral without midline involvement	T3N2b	Bilateral level II-IV neck dissection	50/25	Ipsilateral level III (HRR)	39	Lung metastasis, 56 months later
4	Center 2	central	T3N2b	Bilateral level II-IV neck dissection	50/25	Level II of left neck (HRR)	8	Survival
5	Center 2	unilateral without midline involvement	T4aN3 (ENE)	Bilateral level II-IV neck dissection	50–60/25–30	Ipsilateral level II (HRR)	11	Lost to follow-up

*ENE*, extra nodal extension; *HRL*, high-risk levels (involved nodal levels); *LRL*, low-risk levels (uninvolved nodal levels)

## Discussion

The international guidelines for nodal CTV delineation in the PORT setting have not changed significantly in the last decade [3, 13], regardless of the development of treatment techniques. As diminishing radiation-related toxicities have attracted more attention recently, some doctors have attempted to reduce the intensity of radiotherapy in clinical practice. One possible method is delivering a lower dose to the nodal CTV. By comparing treatment outcomes from the two centers, we found that dose de-escalation to HRL from 60 to 50 Gy significantly increased the risk of regional recurrence. In contrast, a dose of 50 Gy for LRL did not compromise RC of uninvolved nodal levels. Then, dose reduction to the HRL was deprecated, and the LRL was worth considering.

Whether decreasing the dose to the HRL is acceptable has not been validated. The dose required for sterilizing occult disease at the HRL is defined by the volume of microscopic disease and its inherent radiosensitivity [14]. Considering the lower radiosensitivity of neck dissection areas and occult tumor load, the empirical dose used for HRL is 56–60 Gy. However, this prescription was extrapolated from conventional 2D or three-dimensional (3D) radiotherapy experience [15]. As IMRT enables target volumes to receive higher doses, it may be unsuitable. Meanwhile, regional failures are rare in local–regional recurrence [6, 16, 17]. Moreover, owing to the microscopic spread of HRL, a lower dose may be required than that for the primary tumor bed. In center 2, the radiology department adopted a prescription with a lower dose to the HRL, and most patients were considered to have a low risk of regional recurrence in the HRL, i.e., those with stage T1–2N1 tumors and close margin or stage T3–4N0 tumors. Therefore, we analyzed the regional recurrence of dose reduction from 60 to 50 Gy in the HRL using real-world data to investigate whether it was feasible.

It is known that the patterns of regional recurrence could be used to evaluate the appropriateness of PORT: in-field recurrence indicates that nodal target volumes require a higher prophylactic dose, while out-field recurrence is due to the inaccuracy of target delineation [18]. In our study, all regional recurrence events occurred in the field, indicating that CTV delineation is reasonable. However, reduction in the radiation dose delivered to the HRL led to a significant difference in the rate of HRC. A randomized phase III study showed that patients receiving a dose of ≤ 54 Gy had a higher locoregional failure rate than those receiving > 57.6 Gy on first interim analysis [19, 20]. However, the rate of local recurrence was not observed, which may constitute the main cause of locoregional failure. Heterogeneity was also observed among the patients in our study. Nearly half of the patients were treated with chemoradiation in center 1, although the rate of ENE was equal to that in center 2. As more patients were in the late T stage in center 1, more positive therapeutic strategies may have been preferred in clinical practice. Concurrent chemotherapy could help in local–regional control and even increase the OS rate in head and neck squamous cell carcinoma cases, which may contribute to better HRC in center 1. Therefore, less intensive treatment regimens for HRLs and suitable methods for selecting appropriate patient populations require further study.

Moreover, reduction in the dose delivered to LRLs has been recommended in many clinical practices. Our data revealed good RC of LRL with a dose of 50 Gy, including in patients with stage T3–4N0 tumors. As the extent of neck dissection varied, PORT for LRL with and without neck dissection was discussed.

For non-operated LRL (cLRL), 50–54 Gy is typically administered [21]. However, the higher sensitivity of improved diagnostic imaging provides an opportunity for treatment deintensification. Better image quality of CT and MRI helps in the detection of smaller nodal metastasis [22, 23]. When positron emission tomography with Fluor-18-fluorodeoxyglucose (FDG-PET) was introduced, the sensitivity and accuracy of detecting tumor deposits improved. It provides unprecedented accuracy for the staging of neck tumors with a detection threshold between 5 and 10 mm [24, 25]. More microscopic diseases can be diagnosed by combining information from this advanced imaging modality, which means that LRLs contain fewer and smaller subclinical tumor deposits. As a lower dose is required to sterilize a lower number of tumor cells, the maximum size of occult metastasis affects the dose–response relationship and RC. A linear relationship between dose and tumor control was observed when the maximum diameter of occult metastasis was within 3–10 mm. This model suggests that improving every 1 mm of the detection threshold of diagnostic imaging can theoretically reduce the elective dose by 1–2 Gy [26]. Fletcher recommended a postoperative dose of 45–50 Gy in 2-Gy fractions, which achieved high rates of control of surgically undisturbed lymph node metastases [27]. Even when using a dose as low as 36 Gy, the tumor control probability of a subclinical disease of 5 mm was 85% [28]. Some investigators have also found that dose de-escalation in LRL to 40 Gy is feasible, resulting in less toxicity without significant differences in disease control or survival [11, 12, 29]. In our study, only one patient experienced recurrence of cLRL, even after receiving 60 Gy. These results imply that doses of 50 Gy or even lower could be delivered to cLRL.

The delivered radiation dose can be a contentious issue for dissected pathological LRL (pLRL). Including pLRL as a prophylactic dose (50 Gy in 25 fractions or 54 Gy in 30 fractions) has been widely implemented in the UK. Some pLRL may even be included in an intermediate dose of 56–57 Gy, owing to the high possibility of occult lymph node metastases [3, 30]. Our data revealed that 50 Gy was sufficient for pLRL. Furthermore, some researchers have been working on omitting PORT from pLRL in head and neck squamous cell carcinoma cases, which achieved reasonable 5-year rates of RC (93%) and unirradiated neck control (97%) [31]. Subsequently, a prospective DIREKHT trial attempted to omit contralateral pLRL irradiation in a predefined low-risk patient population with head and neck cancer [32]. However, regional failures are rare. A retrospective series of patients with oral cavity cancer experienced no regional recurrence after excluding pLRL from postoperative CTV [33]. However, established studies lack recurrence data for SC in such situations. Therefore, the optimal dose for pLRL requires further study.

Our study also noted that dose-reduction treatment maintained the overall rates of OS, RC, PFS, and DMFS, although it decreased the HRC rate. This may be owing to the higher number of patients with stage II disease in center 2 (27.4% vs. 1.6%), which had patients with better survival outcomes. Moreover, de-escalation of doses to nodal levels could reduce radiation-related toxicities. Lower doses to the swallowing structures decrease the prevalence of dysphagia in patients, which improves patients’ quality of life (QoL), as swallowing dysfunction has a stronger negative impact on QoL than xerostomia [34, 35].

Many studies have focused on definitive radiation, but only a small proportion of them have focused on PORT. To the best of our knowledge, no studies on reducing the HRL dose for PORT have been published. However, in clinical applications, a low risk of regional failure would make radiologists and patients choose a less intense radiotherapy strategy to alleviate toxicities. Our study aimed to determine the possibility of dose de-escalation delivered to the HRL during PORT. However, this study has some limitations. The first is the accuracy of the recurrence site when reconstructed using the original planning scan. Furthermore, after surgery, the nodal levels’ structure may be different from that before surgery. Second, as a retrospective design based on real-world data and characterized by different periods from two centers, selection biases and imbalances existed in the inherent variables. No treatment-related adverse events were observed. The final limitations were the relatively small sample size and implicit heterogeneity among patients.

## Conclusion

This study showed that a reduction in the radiation dose delivered to involved lymph node levels resulted in inferior outcomes in patients with SC using the PORT approach. A lower-dose schedule should be performed within the context of clinical trials, especially for patients requiring a better QoL. Uninvolved levels receiving doses lower than 50 Gy could achieve equal prognoses and less radiation injuries.

## Data Availability

The data that support the findings of this study are available from Department of Radiation Oncology, National Cancer Center/National Clinical Research Center for Cancer/Cancer Hospital, Chinese Academy of Medical Sciences (CAMS) and Peking Union Medical College, but restrictions apply to the availability of these data, which were used under license for the current study, and so are not publicly available. Data are however available from the authors upon reasonable request and with permission of Department of Radiation Oncology, National Cancer Center/National Clinical Research Center for Cancer/Cancer Hospital, Chinese Academy of Medical Sciences (CAMS) and Peking Union Medical College.

## Abbreviations

AJCC: American Joint Committee on Cancer
cLRL: Clinically low-risk levels
CT: Computed tomography
CTV: Clinical target volume
DMFS: Distant metastasis-free survival
ENE: Extranodal extension
FDG-PET: Positron emission tomography with Fluor-18-fluorodeoxyglucose
GTVtb: Gross tumor volume
HRL: High-risk levels
HRC: High-risk levels control
IMRT: Intensity-modulated radiotherapy
LRL: Low-risk levels
MRI: Magnetic resonance imaging
OS: Overall survival
PFS: Progression-free survival
pLRL: Pathologically low-risk levels
PORT: Postoperative radiotherapy
PTV: Planning target volume
QoL: Quality of life
RC: Regional control
SC: Supraglottic cancer
